# Risk factors of postoperative septic cardiomyopathy in perioperative sepsis patients

**DOI:** 10.1186/s12871-022-01714-5

**Published:** 2022-06-22

**Authors:** Yuchang Xin, Ying Ge, Liuhui Chang, Yong Ni, Hairui Liu, Jiang Zhu

**Affiliations:** 1 Department of Anesthesiology, The Second Affiliated Hospital of Soochow University, Suzhou, 215004 People’s Republic of China; 2 Department of Anesthesiology, Sihong People’s Hospital, Suqian, 223900 People’s Republic of China

**Keywords:** Septic cardiomyopathy, Sepsis, SOFA score, Endoscopic surgery, Ejection fraction, Bedside ultrasound

## Abstract

**Objective:**

This study aimed to clarify the relevant risk factors of septic cardiomyopathy (SCM) in perioperative sepsis patients.

**Methods:**

This retrospective study evaluated patients who were diagnosed with sepsis during the perioperative period and postoperatively admitted to the intensive care unit (ICU) in the Second Affiliated Hospital of Soochow University, the First Affiliated Hospital of Soochow University, and the Suzhou Municipal Hospital between January 2017 and November 2020. They were divided into two groups as the septic cardiomyopathy group (SCM group) and the non-SCM group (NSCM group). Factors with *P* < 0.1 were compared between groups and were analyzed by multivariate logistic regression to screen the risk factors of sepsis cardiomyopathy. The area under the receiver operating characteristic (ROC) curve was used to verify the discriminative ability of multivariate logistic regression results. Hosmer-Lemeshow goodness of fit test was used to verify the calibration ability of multiple logistic regression results.

**Result:**

Among the 269 patients, 49 patients had SCM. Sequential Organ Failure Assessment (SOFA) score (adjusted odds ratio [AOR] = 2.535, 95% confidence interval (CI): 1.186-1.821, *P* = 0.000]) and endoscopic surgery (AOR = 3.154, 95% CI: 1.173-8.477, *P* = 0.023]) were identified to be independent risk factors for SCM. Patients with a SOFA score ≥ 7 had a 46.831-fold higher risk of SCM (AOR =46.831, 95% CI: 10.511-208.662, *P* < 0.05). The multivariate logistic regression results had good discriminative (area under the curve: 0.902 [95% CI: 0.852-0.953]) and calibration (c^2^ = 4.401, *P* = 0.819) capabilities. The predictive accuracy was 86.2%. The rates of mechanical ventilation and tracheotomy were significantly higher in the SCM group than in the NSCM group (both *P* < 0.05). The SCM group also had a significantly longer duration of mechanical ventilation (*P* < 0.05) and significantly higher rates of continuous renal replacement therapy (CRRT) and CRRT-related mortality (*P* < 0.05). Further, the total length of stay and hospitalization cost were significantly higher in the SCM group than in the NSCM group (*P* < 0.05).

**Conclusion:**

Endoscopic surgery and SOFA score ≥ 7 during postoperative ICU admission were independent risk factors for SCM within 48 hours postoperatively in patients with perioperative sepsis.

## Background

Sepsis is defined as life-threatening organ dysfunction caused by a dysregulated host response to infection [1]. Sepsis remains a major health problem worldwide, with high morbidity and mortality rates [2]. Septic cardiomyopathy (SCM) is sepsis-associated acute syndrome of cardiac dysfunction unrelated to ischemia with one or more of the main characteristics: (1) left ventricular dilatation with normal- or low-filling pressure; (2) reduced ventricular contractility; and (3) right ventricular dysfunction or left ventricular (systolic or diastolic) dysfunction with a reduced response to volume infusion [3], which is reversible and can be restored at the early stage of sepsis [4]. There is no uniform diagnostic criteria for SCM, and thus, the incidence of SCM varies widely, ranging from 10 to 70% [5]. SCM increases the mortality rate of patients with sepsis by two to three times, making sepsis treatment more difficult and significantly increases the economic burden [6].

The pathogenesis of SCM remains unclear to date. However, previous studies have shown that it is not associated with myocardial ischemia and hypoxia, but rather cardiac inhibition caused by multiple substances such as tumor necrosis factor-alpha and interleukin-1 beta [3, 7]. Latest research show that the pathogenesis of SCM include the following aspects. First, pathogen-associated molecular patterns (lipopolysaccharide) and damage-related molecular patterns (heparin sulfate) can interact with the corresponding receptors (toll-like receptors) and eventually activate nuclear factor-κB, leading to the production of a large number of inflammatory factors that cause cardiac suppression [8]. Second, nitric oxide produced by neuronal and endothelial nitric oxide synthase may cause early cardiac dysfunction, while nitric oxide produced by inducible nitric oxide synthase may cause late cardiac dysfunction, due to mechanisms including changes in preload and afterload, decrease in sensitivity of myocardial filaments to calcium ions, downregulation of adrenaline receptors, and increase in mitochondrial permeability [9]. Third, mitochondrial dysfunction including abnormal mitochondrial structure, mitochondrial DNA damage, increased mitochondrial membrane permeability, and inhibition of cytochrome C oxidase activity, all of which can lead to the increase of reactive oxygen species and abnormal energy metabolism, thus causing myocardial dysfunction [3]. Fourth, calcium dysregulation in cardiomyocytes due to SR Ca^2+^-ATPase (SERCA2) inhibition also plays an important role in SCM [10].

Previous studies have shown that a history of heart failure/coronary heart disease, high lactic acid levels, chronic health assessment system II scores, vasoactive drug use, male sex, and young age were associated with SCM [11]. However, research on relevant risk factors of SCM in perioperative patients is lacking. Clinically, we observed that many patients with SCM underwent endoscopic surgery. Thus, this study aimed to clarify the relevant risk factors of SCM in perioperative sepsis patients. Further, we established a predictive model of SCM within 48 hours after intensive care unit (ICU) admission in surgical patients with perioperative sepsis, to provide a reference for improving the quality of perioperative management in critically ill patients.

## Methods

### Study design and patients

This multicenter retrospective study evaluated patients who were admitted to the ICU after a definitive perioperative diagnosis of sepsis in the Second Affiliated Hospital of Soochow University, the First Affiliated Hospital of Soochow University, and Suzhou Municipal Hospital from January 2017 to November 2020. The inclusion criteria were [1] admission to the ICU after a definitive perioperative diagnosis of sepsis and [2] age ≥ 18 years. The exclusion criteria were as follows: (1) previous heart disease with cardiac insufficiency; (2) preoperative SCM; (3) advanced malignant tumor; (4) acute cardiac dysfunction occurred more than 48 hours after ICU admission; (5) postoperative cardiopulmonary resuscitation; (6) incomplete patient data, including basic data, surgical data, and data during ICU admission; (7) ≥2 surgeries within the same hospitalization.

The Ethics Committee of the Second Affiliated Hospital of Soochow University approved this observational retrospective study and granted a waiver of written informed consent (JD-HG-2020-16). All methods were performed in accordance with the guidelines set forth by the the Second Affiliated Hospital of Soochow University.

### Study protocol

The patients were divided into two groups according to the development of SCM within 48 hours of ICU admission as the SCM group and the non-SCM (NSCM) group. SCM was diagnosed according to the following clinical characteristics: (1) decreased ventricular systolic force; (2) left ventricular dilation under normal or low filling pressure; and (3) right ventricular dysfunction and/or left ventricular dysfunction with reduced infusion response. We used a more stringent diagnostic criteria for SCM according to the following two conditions. First, acute cardiac dysfunction in patients with sepsis or septic shock was confirmed within 48 hours of ICU admission, using one of three methods. [1] Bedside ultrasound performed by the ICU intensive care physician showed a visual decrease in systolic function of the left ventricle or right ventricle(18 out of 49, 36.7%) [12]. [2] Ejection fraction (EF) measured on echocardiography was lower than 50%(24 out of 49, 48.9%). The echocardiography was performed by a professional physician in the Department of Ultrasound. [3] PiCCO monitoring showed a global EF of <15% or a cardiac function index of <3/min (7 out of 49, 14.4%) [13]. Second, acute cardiac dysfunction improved within two weeks after sepsis control (14.20). The results of relevant cardiac inspection data were confirmed by two independent high-qualified doctors in the ICU who did not participate in this study. The presence of acute reversible cardiac function concerns, such as diagnostic classification results, was discussed by another deputy director of the physician.

### Data collection

The basic information on patients, indicators of preoperative status, surgical type, indexes of postoperative ICU admission, and the incidence of SCM within 48 hours of admission to the ICU were collected from the electronic medical record system and the medical record room. Basic information included age, sex, height, weight, body mass index (BMI), history of hypertension, diabetes, and admission route. Intraoperative conditions included preoperative shock, type of operation, method of operation, and source of infection.

ICU admission data included the Sequential Organ Failure Assessment (SOFA) score, initial lactic acid value, maximum lactic acid value, initial platelet value, minimum platelet value, maximum procalcitonin (PCT) value, maximum body temperature, and maximum noradrenaline dose. The timing of SCM (within 48 hours or later) after ICU admission was also determined.

### Variable definitions

Admission was categorized as emergency admission and non-emergency admission. Septic shock was diagnosed based on data from the medical chart and nurse’s chart, with reference to the Third International Consensus Definitions for Sepsis and Septic shock [1]. The types of surgery were classified as emergency surgery or non-emergency surgery. The surgical site was divided into the gastrointestinal tract, liver and gallbladder, urinary system, pelvic cavity, and others. The norepinephrine dosage (ug/kg*min) was determined according to the maximum sustained pump of norepinephrine in the detailed nursing medication record sheet during ICU admission.

### Assessments

SOFA scores were based on the results of laboratory examinations in the ICU. The initial lactic acid value and the maximum lactic acid value during the ICU stay were determined according to the lactic acid value in the blood gas analysis report. The initial platelet value and minimum platelet value during ICU admission were determined.

### Statistical analysis

Normally distributed data were compared using the independent sample t-test, with average ± standard deviation (x ® ± s). Meanwhile, non-normally distributed data were analyzed using two independent samples and presented as the median (quaternary interval) (M [Q1, Q3]). Enumeration data were presented as example (%) and analyzed using either C ^2^ test or Fisher’s exact probability test. Univariate statistical analysis was used to identify potential influencing factors (*P* < 0.1) that were then included in the multivariate logistic regression analysis to screen independent risk factors. The area under the receiver operating characteristic (ROC) curve was used to evaluate the discriminating capability of the results of the multifactor logistic regression equation. The Hosmer-Lemeshow goodness of fit test was used to determine the calibration capability of the results of the multifactor logistic regression equation. All statistical analyses were performed using IBM SPSS Statistics 24. *P* < 0.05 was considered statistically significant. The sample size of this study calculated by the Event per variable (EPV) method was about 240 patients, and the cases we collected were consistent with this data.

## Results

### Patient characteristics

**Fig. 1:**
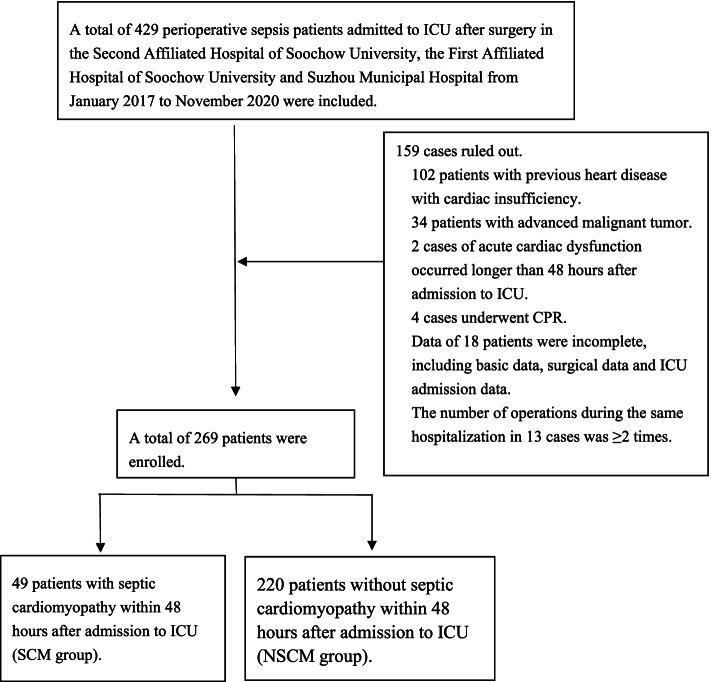
Flowchart of clinical study. Note: AOR: adjusted odds ratio; CI: confidence interval; Tht: The highest temperature; Es: Endoscopy surgery; Nd: Noradrenaline dosage; Mlav: Maximum lactic acid value; Plan: Primary lactic acid number; Tlpv: The lowest platelet value, P value less than 0.05 was statistically significant

A total of 269 patients were included in this study. Among them, 49 and 220 patients were categorized into the SCM group and the NSCM group, respectively. The flowchart of patient inclusion is shown in Fig. 1. Baseline characteristics of patients are shown in Table 1.

**Table 1:** Baseline patient characteristics

Variable	The total number of cases(*n* = 269)	SCM-group (*n* = 49)	NSCM-group (*n* = 220)	*P* value comparing SCM and NSCM
Age, years M(Q1,Q3)	66.0 [52.0, 77.0]	70.0 [52.5, 80.0]	66.0 [51.3, 75.8]	0.247
Male sex	149(55.4)	29(59.2)	120(54.5)	0.555
BMI [Kg/m^2^]	22.8 ± 3.6	23.2 ± 3.5	22.7 ± 3.7	0.390
Medical history				
Hypertension	107(39.8%)	19(38.8%)	88(40.0%)	0.874
Diabetes mellitus	37(13.8%)	7(14.3%)	30(13.6%)	0.905
Emergency admission	185(68.8%)	34(69.4%)	151(68.6%)	0.918
Preoperative shock	111(41.3)	21(42.9)	90(40.9)	0.802
emergency operation	166(61.7)	32(65.3)	134(60.9)	0.591
Endoscopy surgery	76(28.3)	23(46.9)	53(24.1)	0.001
operative region				0.375
Gastrointestinal	153(56.9)	32(65.3)	121(55.0)	
liver and gall	48(17.8)	5(10.2)	43(19.5)	
urinary	55(20.4)	11(22.4)	44(20.0)	
arms and legs	6(2.2)	0(0)	6(2.7)	
Others	7(2.6)	1(2.0)	6(2.7)	
SOFA score M(Q1,Q3)	6[4, 8]	10[8, 12]	5[4, 7]	0.000
Primary lactic acid number M [Q1,Q3] (mmol/L)	1.95[1.1, 3.68]	4.1[2.1, 6.6]	1.8[1.0, 2.9]	0.000
Maximum lactic acid value M [Q1,Q3] (mmol/L)	2.2[1.42, 4.1]	5.2[3.0, 8.25]	2.0[1.3, 3.4]	0.000
Primary platelet value M[Q1,Q3] (*10^9^/L)	176.0[116, 236]	164.0[106.5, 233.5]	184.0[118.3, 239.3]	0.162
The lowest platelet value M [Q1,Q3] (*10^9^/L)	98.0[51.5, 156.5]	33.0[16.5, 95.5]	108.5 [67.5, 172.0]	0.000
Maximum procalcitonin value M [Q1,Q3] (mmol/L)	27.0[6.0, 77.4]	71.5[33.7, 100]	19.0 [4.3, 65.2]	0.000
Noradrenaline dosage M [Q1,Q3] (ug/kg*h)	0.13[0.0, 0.4]	1.1[0.35, 1.69]	0.06[0.0, 0.26]	0.000
body temperature M [Q1,Q3] (°C)	38.3[37.6, 39.0]	38.6[38.0, 39.3]	38.2[37.5, 39.0]	0.058
Mechanical ventilation time [h, M(Q1,Q3)]	12.0 [0, 71.5]	100.0 [11.0, 240.0]	8.0 [0, 29.5]	0.000
Length of ICU stay [d, M(Q1,Q3)]	6.0 [3.0, 10.0]	12.0 [8.0, 19.25]	5.0 [3.0, 8.0]	0.000
Hospitalization cost [yuan, M(Q1,Q3)]	72,486.64 [44,364.5, 114,640.1]	143,472.5 [84,220.1, 242,806.6]	60,049.8 [42,021.0, 96,306.2]	0.000
mechanical ventilation (%)	171(63.6)	39(79.6)	132(60.0)	0.010
CRRT(%)	35(13.0)	18(36.7)	17(7.7)	0.000
tracheotomy(%)	10(3.7)	6(12.2)	4(1.8)	0.000
death(%)	29(10.8)	16(32.6)	13(5.9)	0.000

*Note:* *SCM* Septic cardiomyopathy, *NSCM* Non-septic cardiomyopathy, *P* value less than 0.05 was statistically significant

### Surgical information

The proportion of patients who underwent endoscopic surgery was significantly higher in the SCM group than in the NSCM group (*P* < 0.05). However, there were no significant between-group differences in the incidence of preoperative shock, the proportion of emergency operation, and the site of operation (all *P* < 0.05) (Table 1). There were no significant differences in age, sex ratio, BMI, history of hypertension, and history of diabetes between the endoscopic surgery group and the non-endoscopic surgery group (Table 2).

**Table 2:** Baseline characteristics of patients in endoscopic surgery group and Non-endoscopic surgery group

Variable	The total number of cases(*n* = 269)	Endoscopic surgery (*n* = 76)	Non-endoscopic surgery (*n* = 193)	*P* value comparing SCM and NSCM
**Age, years M(Q1,Q3)**	66.0 [52.0, 77.0]	62.5 [48.5, 75.8]	66.0 [53.0, 77.0]	0.093
**Male sex**	149(55.4%)	40(52.6%)	109(56.5%)	0.568
**BMI [Kg/m**^**2**^**]**	22.8 ± 3.6	23.4 ± 3.6	22.5 ± 3.6	0.090
**Medical history**				
**Hypertension**	107(39.8%)	29(38.9%)	78(40.4%)	0.734
**Diabetes mellitus**	37(13.8%)	14(18.4%)	23(11.9%)	0.163

### ICU stay

As shown in Table 1, the SCM group had a significantly higher SOFA score and maximum norepinephrine dose, as well as a significantly lower minimum platelet value (all *P* < 0.05). The SCM group also included a significantly higher proportion of patients with initial lactic acid value ≥4 mmol/L, maximum lactic acid value ≥4 mmol/L, maximum procalcitonin (PCT) value ≥10 ng/mL, and maximum body temperature ≥ 38.5 °C (all *P* < 0.05).

### Predictors of postoperative SCM

**Fig. 2:**
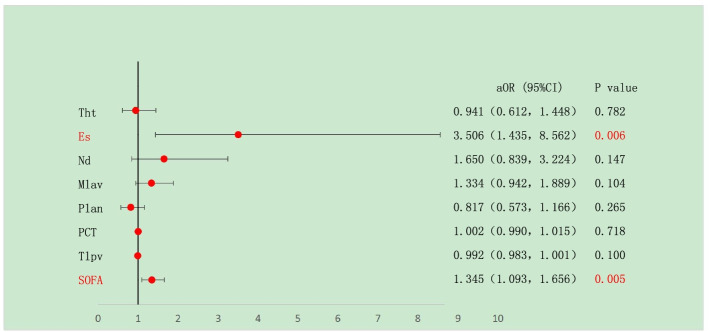
Multivariate correction results of sepsis patients with septic cardiomyopathy

**Fig. 3:**
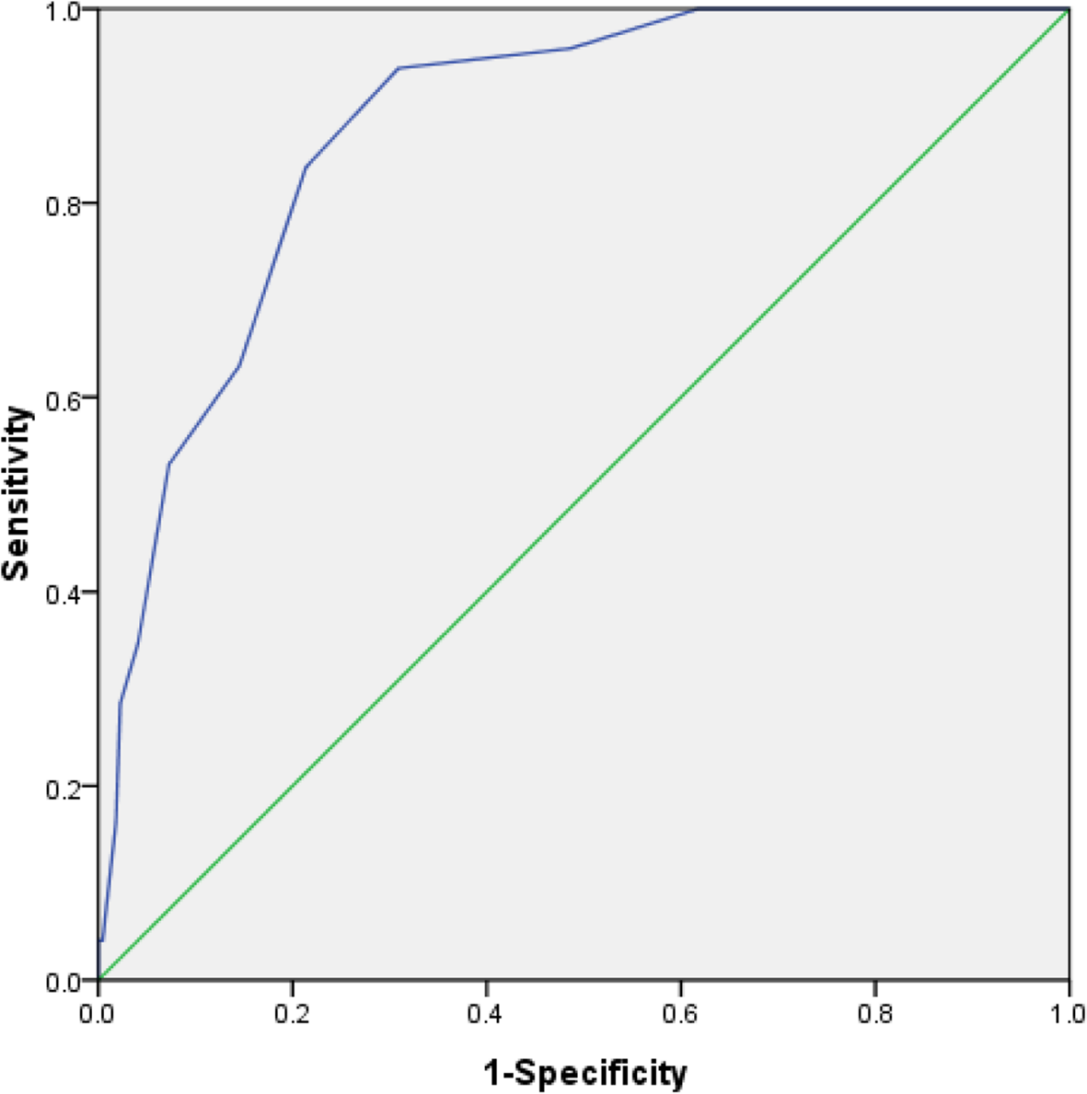
Logistic regression model ROC curve of sepsis patients complicated with septic cardiomyopathy

**Fig. 4:**
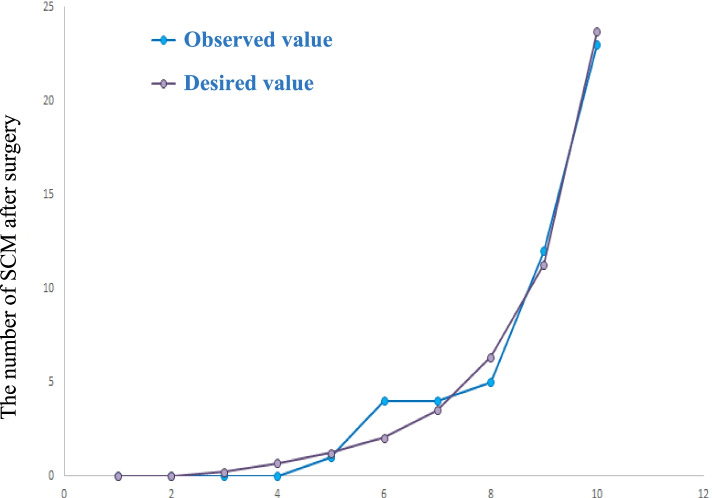
Logistic regression model Hosmer-Lemeshow test of sepsis patients complicated with septic cardiomyopathy

The SOFA score and endoscopic surgery were identified to be risk factors for postoperative SCM in patients with sepsis (*P* < 0.05; Table 3, Fig. 2). The ROC curve indicated that the results of the multifactor logistic regression equation had a good discriminative capability. The area under the curve was 0.912 (95% confidence interval [CI]: 0.8744-0.952, Fig. 3). The Hosmer-Lemeshow test also indicated that the results of the multifactor logistic regression equation had a certain calibration capability, with C^2^ = 3.363 (*P* = 0.888 > 0.05). The predictive accuracy of the results of the multifactor logistic regression equation was 88.6% (Fig. 4).

**Table 3:** Multivariate logistic regression analysis results of sepsis patients complicated with septic cardiomyopathy

Predictive factor	aOR (95%CI)	***P*** value
**SOFA score**	1.345(1.093, 1.656)	.005
**Primary lactic acid number**	.817(.573, 1.166)	.265
**Maximum lactic acid value**	1.334(.942, 1.889)	.104
**The lowest platelet value**	.992(.983, 1.001)	.100
**PCT**	1.002(.990, 1.015)	.718
The highest temperature	.941(.612, 1.448	.782
**Noradrenaline dosage**	1.650(.839, 3.244)	.147
**Endoscopy surgery**	3.506(1.435, 8.562	.006

*Note:* *AOR* Adjusted odds ratio, *CI* Confidence interval, *P* value less than 0.05 was statistically significant

### Relationship between SOFA score and postoperative SCM

The relationship between SOFA score at ICU admission and postoperative SCM was investigated by determining the maximum corresponding truncation value of the Youden index, which was identified by analyzing the ROC curve. Patients were divided into two groups based on the SOFA score as ≥7 points and < 7 points (control). Multivariate logistic regression analysis showed that a SOFA score ≥ 7 was associated with a 34.273-fold higher risk of SCM (adjusted odds ratio [AOR] 34.273, 95% CI: 10.299-114.069, *P* < 0.05) (Table 4). There were no significant differences in age, sex ratio, BMI, history of hypertension, and history of diabetes between the SOFA<7 and SOFA≥7 groups (all *P* > 0.05) (Table 5).

**Table 4:** Influence of SOFA score on septic patients complicated with septic cardiomyopathy

SOFA score	Multiple-factor analysis	
	aOR(95%CI)	*P* value
**<7**	**reference group**	–
**> = 7**	34.273 (10.299-114.069)	0.000

*Note:* *AOR* Adjusted odds ratio, *CI* Confidence interval.”-”: SOFA score < 7 was divided into the reference group

**Table 5:** Baseline characteristics of patients in SOFA<7 group and SOFA≥7 group (According to the analysis of subject operating characteristic curve, the maximum cut-off value of Jorden index was 7)

Variable	The total number of cases(*n* = 269)	SOFA<7 (*n* = 155)	SOFA≥7 (*n* = 114)	*P* value comparing SCM and NSCM
**Age, years M(Q1,Q3)**	66.0 [52.0, 77.0]	65.0 [50.0, 77.0]	67.0 [55.0, 77.0]	0.246
**Male sex**	149(55.4%)	80(51.6%)	69(60.5%)	0.146
**BMI [Kg/m**^**2**^**]**	22.8 ± 3.6	22.7 ± 3.9	22.9 ± 3.3	0.839
**Medical history**				
**Hypertension**	107(39.8%)	61(39.4%)	46(40.4%)	0.869
**Diabetes mellitus**	37(13.8%)	20(18.4%)	17(11.9%)	0.636

### Influence of SCM occurrence on prognosis, length of stay, and medical cost

The prognostic indicators, length of ICU admission, and total cost of treatment were compared between patients in the SCM and NSCM groups. The rates of mechanical ventilation, tracheotomy, and continuous renal replacement therapy (CRRT) were significantly higher in the SCM group than in the NSCM group (*P* < 0.05). The duration of mechanical ventilation was also significantly longer in the SCM group (*P* < 0.05). In addition, the length of ICU admission, total hospitalization cost, and mortality rate were significantly higher in the SCM group than in the NSCM group (*P* < 0.05) (Table 1).

## Discussion

SCM is an sepsis-related acute cardiac insufficiency syndrome unrelated to ischemia [5]. SCM has a high incidence rate and is associated with a long length of hospital stay and high mortality [14]. Importantly, it is a crucial factor affecting the prognosis of perioperative sepsis patients. Sato et al. [14] were the first to report the epidemiological characteristics of SCM, with the hospitalization and ICU duration of SCM patients being significantly longer than those of patients without SCM (median, 43 days vs. 26 days, *P* = 0.04; 9 days vs. 5 days, *P* < 0.01, respectively). The in-hospital and 30-day mortality of SCM patients were 24.1 and 20.7%, respectively. Song et al. [15] also reported an ICU mortality rate of 24.5% for SCM patients. To our best knowledge, this study is the first to report the epidemiological data on postoperative SCM in patients with perioperative sepsis, providing a basis for its early prevention and treatment.

In the current study, the 30-day mortality in the SCM group was as high as 32.4%, and this was significantly higher than in the NSCM group (24.1%). This is also higher than the in-hospital mortality reported in previous studies. Further, the results also showed that SCM significantly increased the utilization rate of mechanical ventilation during ICU hospitalization, as well as the duration of mechanical ventilation, the incidence of tracheotomy, the utilization of CRRT, and the mortality during hospitalization. In addition, SCM significantly increased the length of ICU admission and the total cost of hospitalization.

With the introduction of minimally invasive surgery, endoscopic surgery has become widely used in various clinical specialties [16]. However, we found that most cases of postoperative SCM occurred after endoscopic surgery. Therefore, we conducted this study to determine whether endoscopic surgery is a risk factor for postoperative SCM in patients with sepsis. Multivariate logistic regression analysis confirmed that endoscopic surgery is a risk factor for postoperative SCM in sepsis patients. This could be because of the need to build artificial pneumoperitoneum endoscopy surgery [17] which in turn leads to increased abdominal pressure and increased intra-abdominal infection lesions by factors such as blood flow to the risk of further proliferation. The increased systemic inflammatory response causes enhances the release of inflammatory factors that can inhibit myocardial function and cause heart failure [3]. Another cause is the positioning of patients during endoscopic surgery, which results in the decrease of functional residual capacity. The pneumoperitoneum pressure also increases the airway peak pressure increase and reduces respiratory compliance, leading to inadequate ventilation. Moreover, oxygenation after abdominal surgery might further aggravate the acid-base imbalance [18]. Overall, several factors possibly influence the higher risk of SCM in endoscopic surgery than in open surgery. However, research on the specific mechanism of SCM in endoscopic surgery is still lacking, and further experimental studies are needed to establish the relevant factors or mechanisms.

Our results showed that a SOFA score ≥ 7 was a risk factor for postoperative SCM in patients with sepsis. Patients with a SOFA score ≥ 7 had a 46.831-fold higher risk of SCM than those with a score of <7. A SOFA score of ≥7 indicates significant dysfunction of at least one organ, which indirectly reflects the severity of sepsis at the systemic level. Bergenzaun et al. first demonstrated in a prospective, observational, cohort study that the SOFA score (odd ratio [OR]: 1.6 (95% CI: 1.1-2.3), *P* = 0.018) is an independent predictor of mortality in patients with septic shock [19]. A subsequent prospective study involving 48 sepsis patients confirmed that the SOFA score is a good predictor of mortality in sepsis patients [20]. Similar findings are obtained in this study, but we further showed that a SOFA score ≥ 7 increased the risk of SCM, providing more accurate data for the early prevention and treatment of SCM. However, compared with these two previous studies, this is only a retrospective study, and further prospective studies are needed to validate our findings.

Previous studies have shown that hyperlactic acid is a risk factor for SCM [14]. An elevated lactic acid level is a manifestation of impaired systemic microcirculation and tissue insufficiency. A serum lactic acid value of >4 mmol/L at admission is associated with a high mortality rate, and patients with persistently high lactic acid levels for more than 24 h have poor prognosis [21]. In this study, there was a significant difference in the proportion of patients with high lactic acid value (≥4 mmol/L) between the SCM group and the NSCM group during the screening process, but there was no significant correlation between the lactic acid level and postoperative SCM in the multivariate logistic regression.

The differences in the results between studies may be due to the differences in the included population, and the results of previous studies cannot be generalized to the patient population targeted in this study. Meanwhile, we found that a high SOFA score was an independent risk factor for SCM in patients with sepsis, suggesting that multi-organ failure and lactic acidosis may be the result of systemic dysfunction, rather than the direct cause of SCM.

Age is considered to be an independent risk factor for SCM [9]. Aging-related physiological changes lead to decreased organ function and immune function, and these are usually accompanied by various chronic diseases. Resistance to the systemic inflammatory response in sepsis is weakened, leading to an increased risk of SCM [20]. However, the influence of age is still controversial. Sato et al. [9] reported a significant increase in the incidence of SCM in young and male patients in their study. In the current study, multivariate logistic regression analysis showed that the incidence of SCM was correlated with younger age (OR, 0.96; 95% CI: 0.94-0.99), but the mechanism is still unclear and needs further analysis. Further, unlike previous studies, although patients in the SCM group in our study were older than those in the NSCM group (median age: 70 years vs. 66 years), the difference was not significant. This conflicting findings between the current and previous studies may be due to the retrospective nature of the study and the limitation of the overall sample size. Further investigations are needed to confirm that age as a risk factor for SCM after sepsis.

We also found that the maximum dose of norepinephrine was significantly higher in the SCM group than in the NSCM group, while the minimum platelet value was significantly lower (*P* < 0.05). The proportion of patients with high initial lactic acid value, maximum lactic acid value, maximum PCT value, and maximum body temperature was significantly higher in the SCM group than in the NSCM group (*P* < 0.05). However, multivariate logistic regression analysis showed that these indicators were not independent risk factors for perioperative SCM.

This study has its limitations. First, this is a retrospective study. Although the results indicate a correlation between the risk factors and outcome, the relationship was not causal. Second, because this is a retrospective study, cardiac function was measured using different methods including echocardiography and PiCCO hemodynamic monitoring. This difference can lead to biases in the population that may affect the study results. However, studies have shown the consistency between cardiac ultrasound and PiCCO for cardiac function assessment [13]. Third, a unified diagnostic standard for SCM is yet to be established. At present, SCM is diagnosed according to the following clinical characteristics: (1) decreased ventricular systolic force; (2) left ventricular dilation under normal or low filling pressure; and (3) right ventricular dysfunction and/or left ventricular dysfunction with reduced infusion response. However, we used a more stringent diagnostic criteria for SCM and required the identification of reduced cardiac systolic function to exclude patients with primary cardiac dysfunction. Fourth, because most of our patients had decreased cardiac function on admission, some patients who might have abnormal basic cardiac function combined with sepsis were included in the SCM group. To reduce the influence of this limitation, we included improvement of cardiac function after sepsis treatment in the diagnostic criteria for SCM. Finally, only 269 patients were included in this study. From the perspective of risk factors, a larger sample size is needed to obtain more reliable results. Further prospective studies are needed to verify the results of this study.

## Conclusion

Endoscopic surgery and a SOFA score ≥ 7 during postoperative admission to the ICU were independent risk factors for SCM within 48 hours postoperatively. For sepsis patients who need surgery, open surgery may lower the occurrence of SCM. Further, patients with a SOFA score of ≥7 points on ICU admission need to be closely monitored for SCM to provide timely diagnosis and treatment.

## Data Availability

All data generated or analysed during this study are included in this published article.

## Abbreviations

SCM: Septic cardiomyopathy
ICU: Intensive care unit
NSCM: Non-septic cardiomyopathy group
SOFA: Sequential Organ Failure Assessment
CI: Confidence interval
CRRT: Continuous renal replacement therapy
SERCA2: SR Ca^2+^-ATPase
BMI: Body mass index
PCT: Procalcitonin
EF: Ejection fraction
ROC: Receiver operating characteristic
EPV: Event per variable
AOR: Adjusted odds ratio
OR: Odd ratio
